# Evolution of Metastable Defects and Its Effect on the Electronic Properties of MoS_2_ Films

**DOI:** 10.1038/s41598-018-24913-y

**Published:** 2018-04-30

**Authors:** M. Precner, T. Polaković, Qiao Qiao, D. J. Trainer, A. V. Putilov, C. Di Giorgio, I. Cone, Y. Zhu, X. X. Xi, M. Iavarone, G. Karapetrov

**Affiliations:** 1 Department of Physics, Drexel University, Philadelphia, PA 19104 USA; 2 Department of Physics, Temple University, Philadelphia, PA 19122 USA; 3 Department of Condensed Matter Physics and Materials Science, Brookhaven National Laboratory, Upton, NY 11973 USA; 4 Institute of Electrical Engineering, Slovak Academy of Sciences, Bratislava, 841 04 Slovak Republic; 5 Department of Physics, University of San Francisco, 2130 Fulton St., San Francisco, CA 94117 USA; 6 Present Address: Institute for Physics of Microstructures RAS, Nizhny Novgorod, GSP-105 603950 Russia; 7 Present Address: E.R. Caianiello Physics Department and NANOMATES, Research Centre for Nanomaterials and Nanotechnology, University of Salerno, Fisciano (SA), Italy

## Abstract

We report on structural and electronic properties of defects in chemical vapor-deposited monolayer and few-layer MoS_2_ films. Scanning tunneling microscopy, Kelvin probe force microscopy, and transmission electron microscopy were used to obtain high resolution images and quantitative measurements of the local density of states, work function and nature of defects in MoS_2_ films. We track the evolution of defects that are formed under heating and electron beam irradiation. We observe formation of metastable domains with different work function values after annealing the material in ultra-high vacuum to moderate temperatures. We attribute these metastable values of the work function to evolution of crystal defects forming during the annealing. The experiments show that sulfur vacancies formed after exposure to elevated temperatures diffuse, coalesce, and migrate bringing the system from a metastable to equilibrium ground state. The process could be thermally or e-beam activated with estimated energy barrier for sulfur vacancy migration of 0.6 eV in single unit cell MoS_2_. Even at equilibrium conditions, the work function and local density of states values are strongly affected near grain boundaries and edges. The results provide initial estimates of the thermal budgets available for reliable fabrication of MoS_2_-based integrated electronics and indicate the importance of defect control and layer passivation.

## Introduction

Transition metal dichalcogenides (TMDs) and other 2D materials are currently receiving wide attention from the scientific community due to their unique physical^1^ and chemical^2^ properties. The prototypical TMD material MoS_2_, shows great promise for next generation logic circuits^3^, optoelectronics^4^ and sensing^5^. Many of these applications require high-quality crystals with small defect concentrations that are stable under thermal^4^ and electromagnetic radiation^6^. While abundant in nature as Molybdenite, MoS_2_ can be also fabricated by various synthetic growth methods^7–11^, resulting in different concentrations of defects^12^. These defects are known to have great impact on optical, electronic and magnetic properties^13–19^ and as such, characterization of defect formation and dynamics is important for further development of applications based on MoS_2_.

Previous experiments on defects and their impact on electronic structure of monolayer MoS_2_ were carried out primarily by scanning tunneling microscopy and spectroscopy and photoluminescence spectroscopy^20–22^. Changes of surface structure and tunneling spectra was observed at edges and grain boundaries with presence of mid-gap electronic states^23,24^. This led to observation of both p-type and n-type doping with varying contact resistance in molybdenite and synthetic MoS_2_^12,15,16,19,22^. Strong interactions with the substrate could also induce local spatial modulation of the density of states via local strain or local charge accumulation^25^. It was found that the work function of MoS_2_ is dependent on growth substrate and presence of contaminants on the MoS_2_ surface^18^. Recent experiments carried out using transmission electron microscopy show that sulfur vacancies exhibit non-trivial dynamics, in which a single point defect can migrate through the crystal lattice. Collective mobility of many point defects leads to formation of extended line defects^26^ and other mobile complex defect structures^27^, which have tendency to migrate through the crystal and annihilate at grain boundaries or crystal edges^28^. Prolonged exposure to electron beam irradiation in ultra-high vacuum conditions has been also shown to cause complete dissociation of MoS_2_, with sulfur atoms evaporating from the material, decoupling the molybdenum sheet and allowing it to recrystallize into its native *bcc* crystal structure^28^. Understanding of these processes in synthetically grown MoS_2_ is important for design of thin film MoS_2_ electronic devices, particularly for device stability and the choice of material for contacts and gates^16^, where homogeneity and predictable behavior of the work function and doping is crucial. Thermal budgets for integration of MoS_2_ with other electronic materials into specific devices would also be affected by proper estimates of the activation energies for defects’ formation and their time evolution.

Here we present studies of structural and electronic properties of chemical vapor deposited (CVD) MoS_2_ mono- and few-layer crystalline films using combination of scanning tunneling microscopy (STM) and spectroscopy (STS), Kelvin probe force microscopy (KPFM), and transmission electron microscopy (TEM). Firstly, using STM and STS we identify the equilibrium structure, grain boundaries and edge defects and their electronic signatures. Point defects are associated with interstitial molybdenum atoms and sulfur vacancies, with localized electron-like and hole-like states, respectively. The grain boundary defects, on the other hand, result in decrease in the semiconducting gap due to local strain effects. At the edges of MoS_2_ film the gap vanishes and conducting states appear parallel to the film’s edge. Secondly, we observe changes in work function with the number of MoS_2_ layers that follow a trend consistent with findings on exfoliated crystals^18^. Thirdly, we take unique snapshots of time evolution of the spatial redistribution of defects induced during *in-situ* low temperature annealing in ultra-high vacuum (UHV) conditions. Quantitative KPFM measurements of the work function spatial profile in UHV show existence of metastable surface states that gradually form domains and migrate to the grain boundaries where they annihilate. Time-dependent TEM data confirm that the origin of lower metastable values of the work function of CVD-grown MoS_2_ are related to creation of n-doped regions on the surface due to formation and movement of sulfur vacancies.

As the metastable surface potentials are generated in conditions that are common in device fabrication (i.e. low-temperature annealing), their presence might be considered detrimental to the stable performance of MoS_2_-based electronic devices. Therefore, the importance of passivation of MoS_2_ surface by different methods like chemical stabilization with thiol molecules^29^ might be considered.

## Results

### Equilibrium Defects and their Electronic Signatures

Scanning tunneling microscopy and spectroscopy measurements provide information on local structural and electronic properties of the material. In our case we investigate mono- and few-layer single crystalline MoS_2_ films grown on highly ordered pyrolytic graphite (HOPG) using CVD method. In the case of monolayer thick films grown in the regime of van der Waals epitaxy, the local changes in the surface potential of the substrate could significantly perturb the electronic structure of the dichalcogenide film^24,30^. Graphite substrates are found to be by far less invasive and more electronically uniform, making it possible to access the intrinsic low-energy spectrum of quasiparticle excitations in 2D materials via STM^31,32^. Few layer MoS_2_ films were grown on HOPG substrates using ambient pressure CVD technique with solid MoO_3_ and S precursors^10^. This technique yields highly crystalline, stacked single-layer MoS_2_ domains^22^.

**Figure 1:**
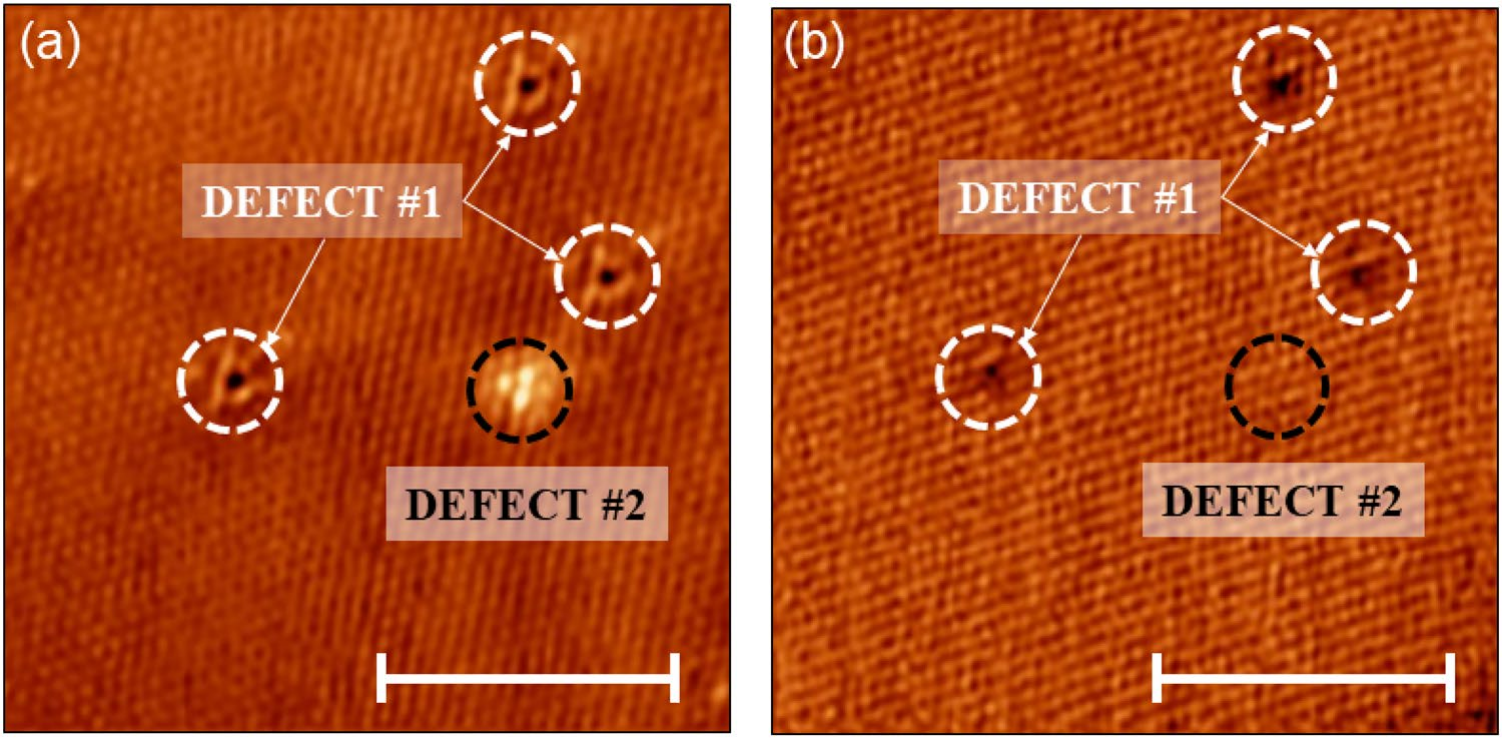
Energy dependent STM on defects. (**a**) and (**b**) show the STM topography of the same area of monolayer MoS_2_ measured at −0.7 V and +0.7 V, respectively (I = 110 pA and 130 pA, respectively). Two types of defects are displayed: defect **#1** outlined with a white circle and defect **#2** outlined in a black circle. All scale bars represent 5 nm.

The films prepared *ex-situ* were degassed at 300 °C from 2.5 to 10 hours in UHV to obtain clean surfaces suitable for STM investigation, and subsequently moved to the STM chamber without breaking the vacuum at room temperature. Figure 1 shows typical atomically resolved STM topography of the top sulfur layer with two different types of defects present as either a dark spot (defect **1**) or three bright adjacent sulfur atoms in a triangular pattern (defect **2**) outlined by a white and black dashed circle, respectively. STM does not have chemical sensitivity, however the nature of the defects can be inferred by their electronic properties. For example, defect **2** is apparent when scanning with −0.7 V, but not when scanning at +0.7 V, in the empty-state regime. This behavior indicates an electron donor nature of the defect. Previous reports using DFT and STM on defects in TMDs have shown that defects of this kind can be explained by an extra transition metal atom in the van der Waals gap beneath the top layer^33,34^. Thus, we conclude that this kind of defect is likely due to an extra interstitial molybdenum atom. The presence of defect **1**, on the other hand, is not dependent on the energy and instead is likely a topographic feature corresponding to a sulfur vacancy in the top sulfur layer.

**Figure 2:**
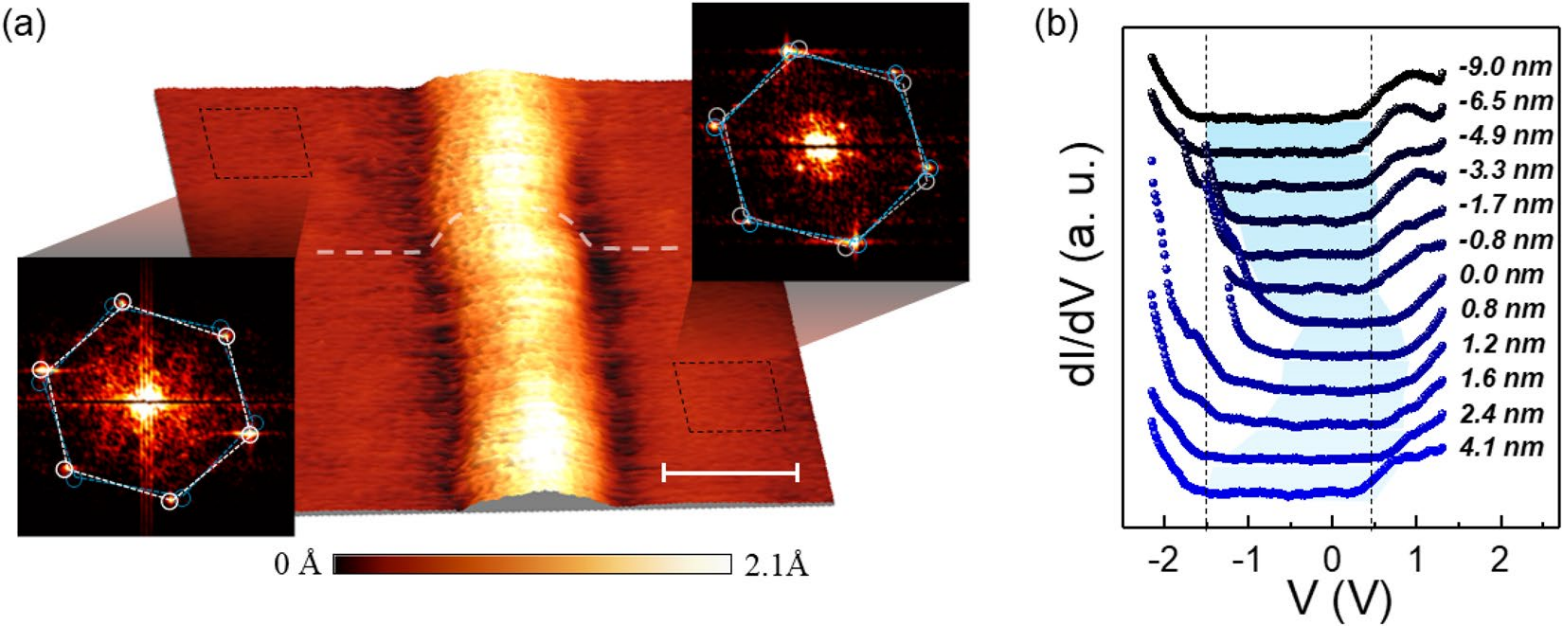
STM/STS across a grain boundary. (**a**) A 3D representation of an STM topography illustrating a grain boundary between two monolayer MoS_2_ domains (V = −1.5 V, I = 100 pA). The Fast Fourier Transform taken from atomic resolution images from the left and right side of the grain boundary are shown as insets on their respective sides. The blue and white hexagons outline the lattice orientations of the right and left domains, respectively. The scale bar represents 5 nm. (**b**) evolution of the tunneling spectra with crossing the grain boundary where the spectrum from top to bottom are taken from left to right over the grain boundary in (**a**) (V = 1.5 V, I = 30 pA). Each spectrum is labeled by its distance from the center of the grain boundary starting from the left side (negative offset) and going to the right side (positive offset) of the grain boundary. Tunneling spectra are taken along the white dashed line in (**a**).

Along with atomic scale defects, the MoS_2_ films exhibit extended defects such as the grain boundary depicted in Fig. 2. The grain boundary appears as a protrusion at negative energies and a depression at positive energies indicating that there is a strong effect on the local electronic density of states. This phenomenon has been attributed to charge transfer and strain in the crystal at the interface between two misoriented grains^21,33^. The relative misorientation between the two grains has been determined to be 6° by comparing atomic resolution topography on the left and right side of the interface, respectively. The electronic effect of such a structure is elucidated by taking spatially resolved scanning tunneling spectroscopy along a profile across the grain boundary (Fig. 2b). The tunneling spectra shown in Fig. 2b from top to bottom are taken across the grain boundary from left to right. It is clear that the grain boundary produces a reduction in the gap mostly from the valence band edge shift consistent with previous reports of local strain and charge transfer effects^22^. For the case of a 6° relative crystallographic angle the gap increases from ~1.0 eV on the grain boundary to its expected monolayer gap of ~2.0 eV around 4 nm away from the center.

**Figure 3:**
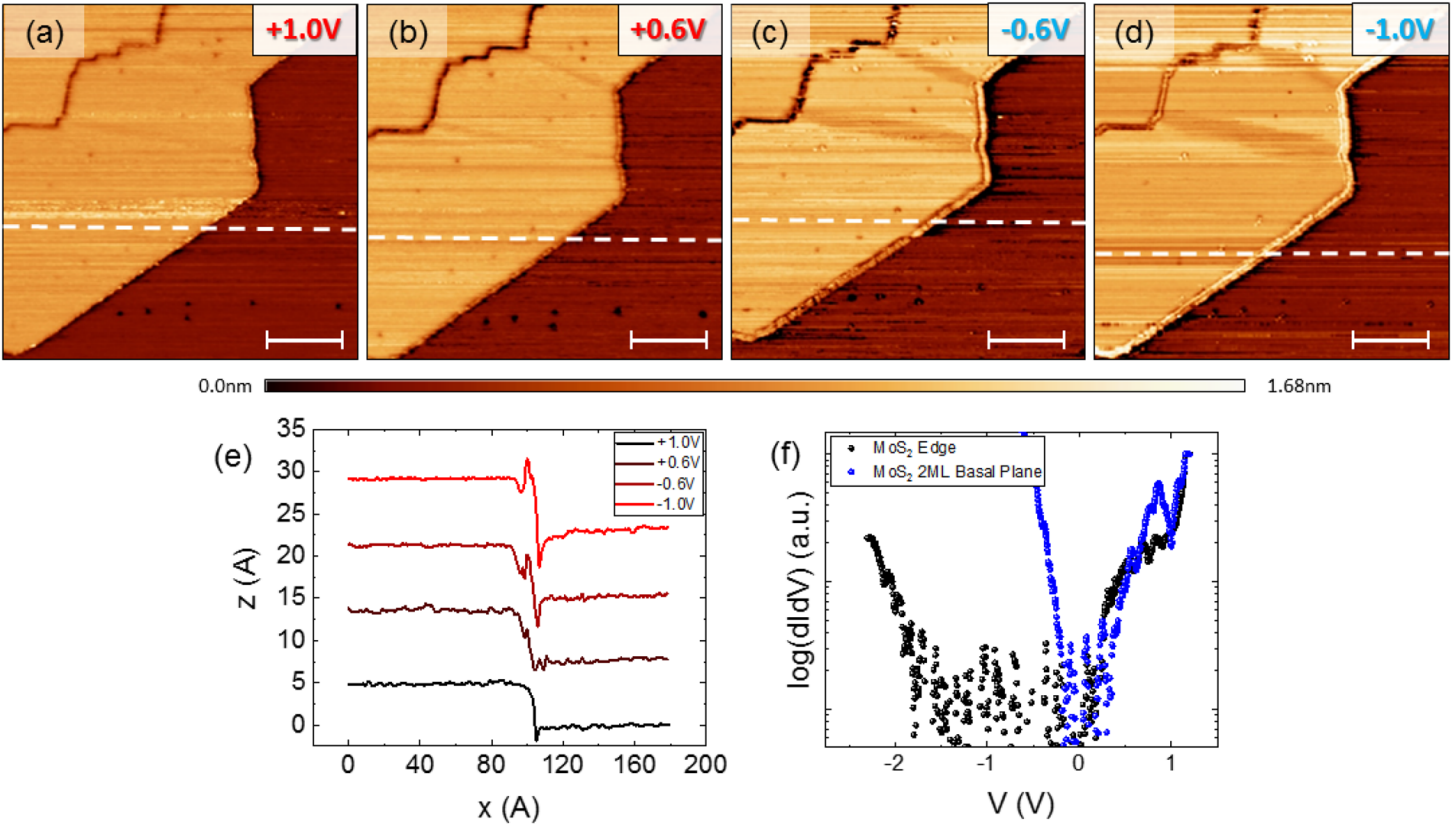
STM/STS on the edge of a bilayer MoS_2_ island. (**a**–**d**) Energy dependent STM topography of the edge of a bilayer island sitting on monolayer MoS_2_ (with tunneling bias displayed in the upper right corner of each topography (I = 30 pA, 30 pA, 60 pA and 80 pA, respectively). All scale bars represent 50 nm. (**e**) Topographic line profiles as a function of energy taken along the dashed line in (**a**–**d**). Representative STS taken on the basal plane (black) and the edge (blue) of the bilayer island shown in (**a**) (V = 1.5 V, I = 30 pA).

Reduced coordination number of the edge atoms of the film results in modification of the band structure of the MoS_2_ in the vicinity of film’s edges. Molybdenum disulfide exhibits confined metallic states which run parallel to the edges of the film^20,35,36^. Figure 3 shows the edge of a bilayer MoS_2_ crystal on a monolayer grain and the effect of the scanning bias voltage on its apparent structure. Figure 3e shows a line profile taken across the edge of the bilayer island as a function of energy. The bilayer edge becomes enhanced as the tunneling energy becomes more negative. The tunneling spectra shown in Fig. 3f display a reduction of the band gap from ~1.9 eV on the bilayer basal plane to ~0.5 eV at the bilayer edge, in good agreement with previous findings^37,38^.

### Metastable defects and their time evolution

In many dichalcogenides a relatively small amount of external (activation) energy could create defects that are metastable in time at room temperature. This activation energy could be delivered in many ways, most common ones being by thermal heating, electron irradiation^26^, or photon illumination^39^. The creation and annihilation of defects could be a reversible process depending on defect type, concentration, and the energy landscape for the metastable state of the lattice to migrate back to its original equilibrium state. Here we employ STEM and KPFM to track the evolution of defect states on the surface of mono- and few-layer MoS_2_ films. The techniques allow us to correlate structural changes with electronic evolution by monitoring the atomic lattice and the work function on the surface of the film in real time.

**Figure 4:**
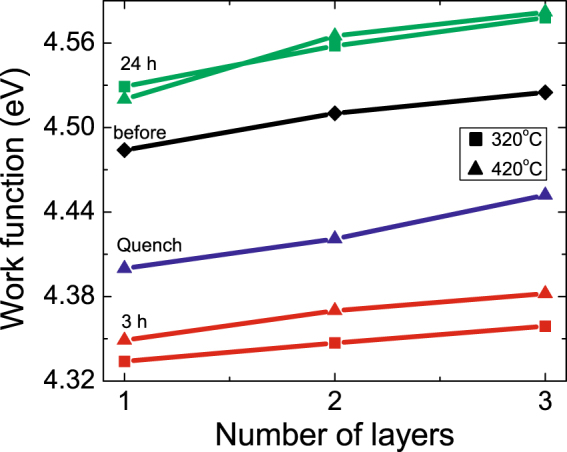
Evolution of the work function of MoS_2_ film ${\varphi }_{Mo{S}_{2}}$ before and after annealing for mono-, two-, and three-layer films. The annealing was performed at 320 °C (□) and 420 °C (Δ) and measured 3 hours and 24 hours after reaching the room temperature. The blue Δ show the values of the work function when the sample was rapidly quenched from 420 °C to room T using liquid nitrogen. The values are compared to equilibrium values of the work function at room temperature (black diamonds).

We employ non-contact atomic force microscopy and frequency modulated Kelvin probe force microscopy to simultaneously determine topography and spatial distribution of surface potential of CVD -grown MoS_2_ on HOPG. To determine work function of MoS_2_ from measurements of contact potential difference (*V*_*CPD*_), we calibrate our AFM tip using the known work function of HOPG *ϕ*_*HOPG*_ = 4.6 eV^40^. To eliminate effects of surface contaminants, annealing was performed in the AFM microscope chamber at 320 °C and p = 2×10^−8^ Torr for 12 hours and the samples were let to slowly cool down to room temperature and equilibrate at p = 1.2×10^−10^ Torr. This process took between 24 to 48 hours. The KPFM experiments provide contact potential difference between the tip and the sample. The work function is determined from the relation:

$${\varphi }_{Mo{S}_{2}}={\varphi }_{HOPG}-e\cdot ({V}_{CPD}^{HOPG}-{V}_{CPD}^{Mo{S}_{2}}),$$

where ${V}_{CPD}^{HOPG}\,and\,{V}_{CPD}^{Mo{S}_{2}}$ are the contact potential differences between the tip and the HOPG substrate, and the tip and the MoS_2_ film, respectively. The *ϕ*_*HOPG*_ = 4.6 eV is the work function of the HOPG substrate^41^. From the measurement under these equilibrium conditions the thickness-dependent work function of MoS_2_ was determined to be ${\varphi }_{Mo{S}_{2}}^{1L}$ = 4.56 ± 0.05 eV for a single-unit cell thick film, ${\varphi }_{Mo{S}_{2}}^{2L}$ = 4.57 ± 0.04 eV for a two-unit cell, ${\varphi }_{Mo{S}_{2}}^{3L}$ = 4.59 ± 0.02 eV for a three-unit cell thick MoS_2_ film (Fig. 4). This is in good agreement with previous UHV KPFM measurements performed on exfoliated MoS_2_ on Si substrates^18^. Small systematic deviation can be attributed to effects of strain from substrate, reported to cause changes as large as 40 meV in the value of the work function of MoS_2_^18^.

**Figure 5:**
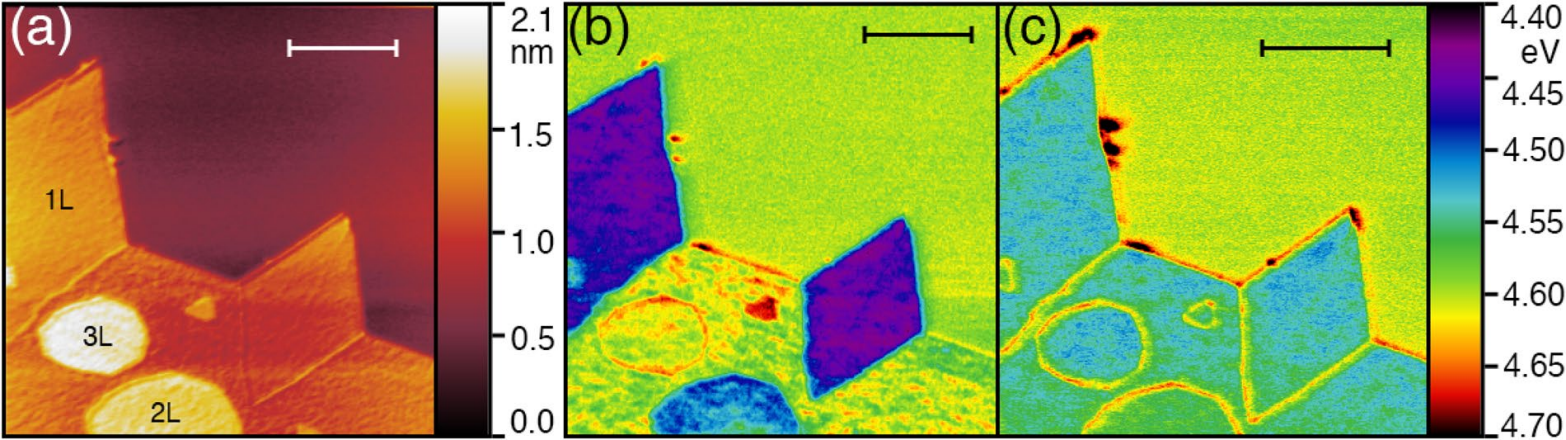
Map of changes in work function ${\varphi }_{Mo{S}_{2}}$ after annealing. Topography image (**a**) with map of work function 3 hours (**b**) and 24 hours (**c**) after annealing. Inset scale bars represent 500 nm in all three figures.

The metastable states of the work function were accessed by subsequent annealing and cooling cycles with immediate KPFM measurements at room temperature. After the first annealing cycle of the sample at 300 °C for 12 hours in UHV conditions (2×10^−8^ Torr) and subsequent cooling to room temperature, non-trivial time evolution of the work function of MoS_2_ could be observed. A typical case is presented in Fig. 5. The MoS_2_ crystalline film consists of large monolayer grains separated by grain boundaries, as well as two-layer (2 L) and three-layer (3 L) islands (Fig. 5a). KPFM images are taken soon after the cool-down: immediately once temperature drops to T = 21 °C (Fig. 5b) and 24 hours later (Fig. 5c). It can be observed, that before reaching a state with homogeneous distribution of work function in the grains in Fig. 5c, the sample goes through a long-lived metastable state with work function in different grains of the film strongly deviating from their equilibrium values. If one tries to subsequently repeat the annealing cycle at the same temperature, the metastable state of the work function can not be reached. After the second annealing at the same temperature the sample returns to its state with equilibrium value of work function immediately after cooling to room temperature. In order to induce the long-lived metastable state again, the sample needs to be annealed at higher temperature (of at least 340 °C in our case). This indicates that the metastable state is caused by mechanism that is not fully reversible. This makes the reversible transition from insulating semiconducting trigonal prismatic 2H-MoS_2_ to metallic octahedral 1T-MoS_2_^42,43^ an unlikely cause of this non-equilibrium behavior. This conclusion is further supported by the fact that the characteristic metallic behavior of 1T-MoS_2_ was not observed by scanning tunneling spectroscopy and that for the 1T-MoS_2_ phase to stabilize in synthetic MoS_2_, electron doping is necessary, either by electron irradiation, or by intercalation with donor atoms, such as lithium^43–46^.

The origin of the metastable behavior of the work function is most likely due to thermally activated migration of MoS_2_ crystal lattice defects, which are known to cause changes in electronic properties of the material by introduction of electronic mid-gap states^17–19,47^. Specifically, sulfur-deficient layers with dangling molybdenum bonds tend to induce gap states close to the conduction band edge^13,19,22^. Presence of these states causes n-type behavior and effectively lowers the measured work function as seen in Fig. 5b (the colder/blue colors in two diamond-shaped islands compared to equilibrium state in Fig. 5c). Considering the large time scales of the relaxation process and the fact that the sample is kept in ultra-high vacuum conditions, the dynamics of these defects is the prime candidate for the temperature-driven metastable values of the work function. This scenario of thermally activated evolution of sulfur vacancies is further corroborated by energy considerations: the predicted energy needed to form a molybdenum vacancy in MoS_2_ is 6.2–6.9 eV, while a single sulfur vacancy costs only 2.1–2.6 eV, enabling sulfur dynamics in MoS_2_ at these low annealing temperatures^12,13,19^.

**Figure 6:**
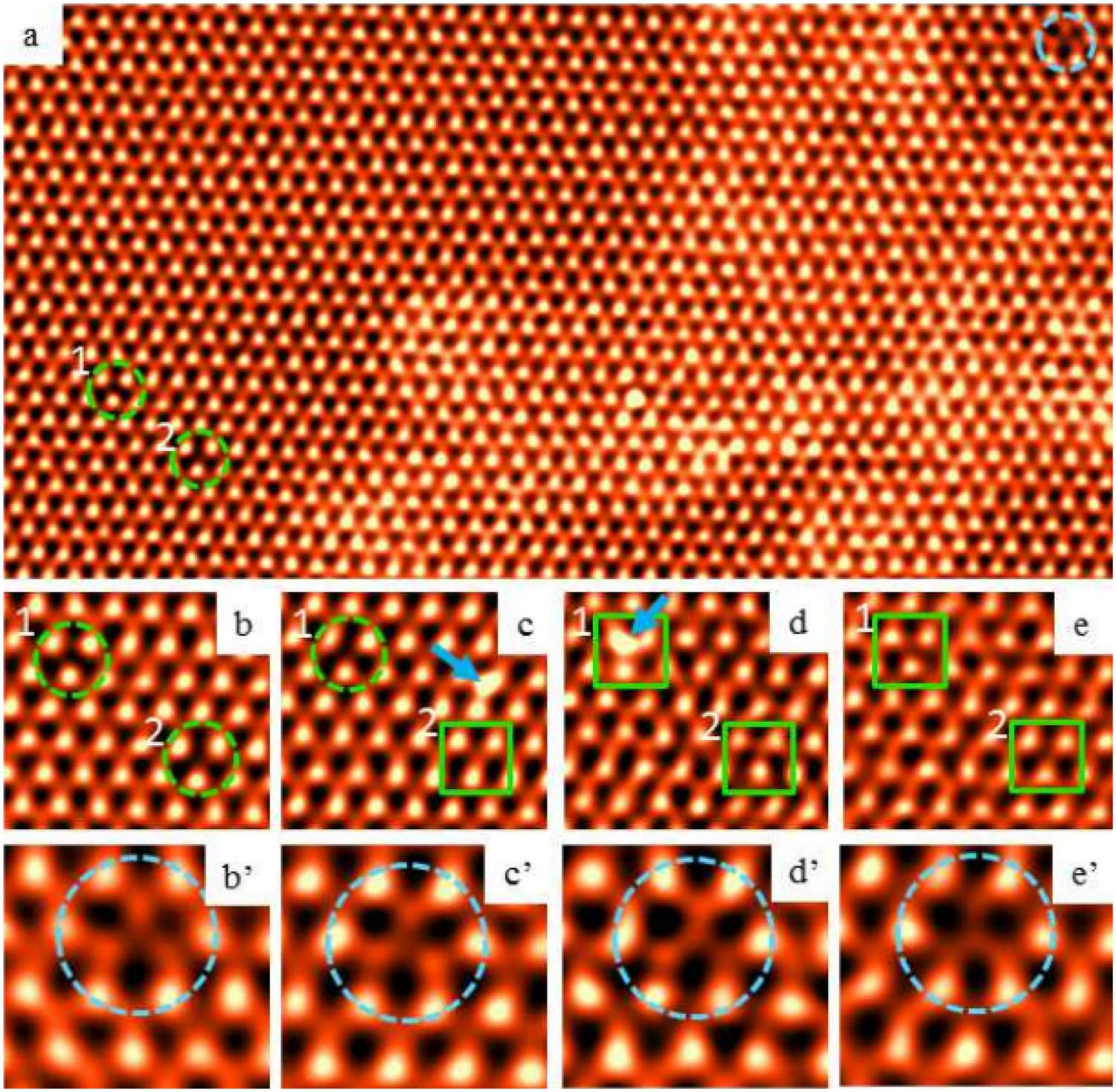
ADF-STEM images showing S vacancy replenishment. (**a**) is an overview of the area of MoS_2_ films that was repetitively scanned, taken at the beginning of the continuous acquisition. Blue dotted circle indicates a Mo deficient site, green dotted circles indicate two S vacancy sites. (**b**) is cropped from (**a**) to show S vacancy sites 1 and 2 at the beginning of acquisition (t = 0). (**c**) is acquired 225 s after (**b**), showing an adatom (blue arrow) and re-occupied site (green square). (**d**) is acquired 300 s after (**c**), showing an adatom at site 1. (**e**) is acquired 225 s after (**d**), showing sites 1 and 2 both occupied by an adatom. (b’–e’) are taken from the same images as (**b**–**e**), respectively, showing Mo deficient site that did not change during acquisition.

To confirm our proposed scenario for the dynamics of sulfur vacancies in CVD -grown MoS_2_ we have performed atomically resolved studies of sulfur vacancy dynamics using annular dark field (ADF) scanning transmission electron microscopy (STEM). A series of ADF images were taken by repetitively scanning over the same area. Figure 6a is an ADF image taken at the beginning of the repetitive acquisition sequence. One notices two sulfur vacancies (marked by green dashed circles) that are a few unit cells away from each other. In the upper right corner of the image one finds a molybdenum-deficient site that we use as a reference position marker of the scanning field of view. The right half of this area is covered by amorphous impurities that could be the source of foreign atoms (Mo and S atoms). Figure 6(b) is cropped from Fig. 6(a) and shows zoom-in of the sulfur vacancies. In Fig. 6(c), that was acquired 225 s after Fig. 6(b), we notice that the lower right S vacancy site got re-occupied by an adatom (green square), and a new impurity atom suddenly appeared (blue arrow). Next, Fig. 6(d), acquired 300 s after the image 6(c), shows that the impurity atom travelled to the upper left sulfur vacancy site (blue arrow) under the influence of the scanning electron beam irradiation. And finally, both vacancy sites were re-occupied by adatoms, as shown in Fig. 6(e), that was taken 225 s after Fig. 6(d). Figure 6(b’–e’) were cropped from the same images as Fig. 6(b–e), respectively, to show that the molybdenum-deficient site remained unoccupied and static during the continuous electron beam irradiation during these 750 s of scanning. On the other hand, sulfur vacancy sites during this time were mobile and easily annihilated. The ADF-STEM results confirm that the origin of lower metastable values of the work function in CVD-grown MoS_2_ are most likely due to formation of n-doped regions on the parts of the MoS_2_ surface rich with sulfur vacancies.

Once formed, these vacancies have been observed to diffuse throughout the lattice and coalesce, forming extended line defects^26,28^ and mobile defect complexes, that could eventually get absorbed into crystal grain boundaries or layer edges^27^. Indeed, we observe that the domains of non-equilibrium work function always terminate at either a grain boundary or layer edge. The general mechanism explaining the time evolution of the metastable states is thermally activated movement of the sulfur vacancies formed during the annealing process. The vacancies slowly diffuse upon cooling, coalesce together and they finally annihilate at boundaries/edges. This time evolution continues until the system reaches equilibrium defect concentration, or until the energy barrier for diffusion becomes too large at room temperature.

**Figure 7:**
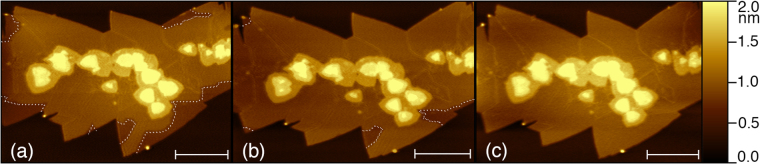
AFM images of MoS_2_ at constant tip-sample voltage (**a**) 3 hours after annealing, (**b**) 5 hours after annealing, and, (**c**) 7 hours after annealing. Domains of different contact potential can be observed to slowly contract until the sample reaches a homogeneous state with ${\varphi }_{Mo{S}_{2}}\approx$ 4.6 eV. The domain walls in (**a**) and (**b**) are highlighted by dotted line. Scale bars correspond to 500 nm.

The time evolution of the domain wall (separating areas with different work functions) movement during post-anneal equilibration is shown in Fig. 7. In Fig. 7(a) one could see the domain wall boundary separating the areas of single unit cell MoS_2_ different work function near the outer edges of the layer. As time evolves the boundary slowly propagates to the edge of the layer in Fig. 7(b), and finally reaches the edge of the first layer. The overall relaxation process of the work function took approximately 7 hours after the sample was brought back to room temperature. From the recording of this time evolution we extract the quantitative information about the energy barriers in the sample. We can approximate the diffusion coefficient for domain movement by

$$D\approx \frac{{r}^{2}}{t},$$

where *t* is the time needed for a domain wall to travel a perpendicular distance *r*. Using this approximation, we get a diffusion coefficient *D*(300 K) ≈ 5×10^−15^ cm^2^/s. Assuming that the domain wall motion is dominated by self-diffusion of sulfur, we can use the Arrhenius relation to extract the associated activation energy. With a previously measured value of *D*(1300 K) = 3.2 × 10^−7^ cm^2^/s^48^, the activation energy was determined to be ${E}_{A}\mathrm{=390}\times {k}_{{\rm{B}}}log(\frac{D(\mathrm{300}\,K)}{D(\mathrm{1300}\,K)})\approx 0.6$ eV. This value is much smaller than the estimated energy barrier for *creating* a new sulfur vacancy (approximately 2.4 eV^12,13,19^), and consistent with previous experiments estimating the energy barrier for *diffusion* of sulfur vacancies measured via changes in conductivity^49^, direct TEM observations^26^, and theoretical calculations of dramatic reduction of the energy barrier for vacancy diffusion within a MoS_2_ layer with high concentration of pre-existing defects^26^.

**Figure 8:**
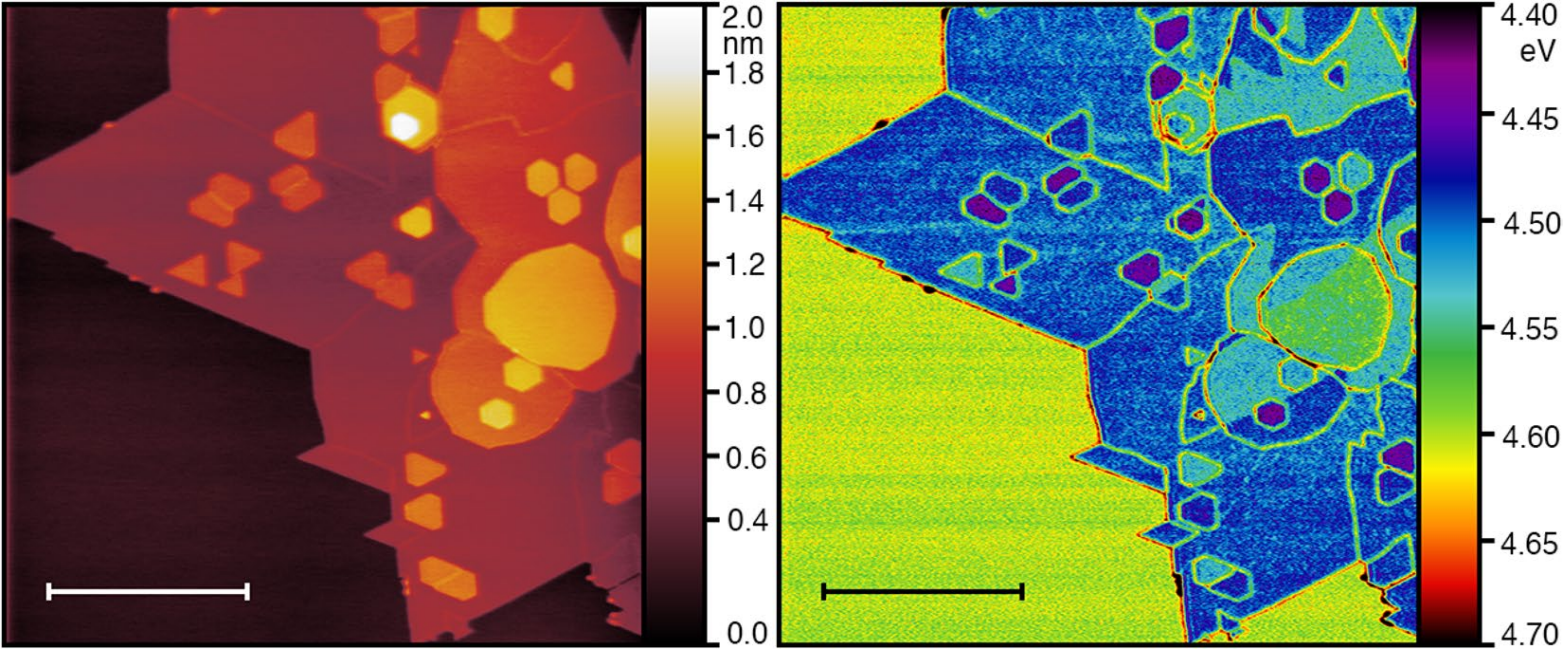
Topography (left) and work function map (right) of a MoS_2_ film after fast cooling from 300 °C to room temperature. Stable domains with different work function on 2nd and 3rd layers are evident. The first layer (with exception of grain boundaries) is homogeneous with work function of roughly 4.5 eV. Scale bars indicate 1 *μ*m.

To explore the effect of temperature on the relaxation of the metastable states to equilibrium, we measured spatial distribution of work function on a sample that was annealed at 300 °C in UHV for 6 hours and then rapidly quenched down to room temperature within 15 minutes by cooling the sample using flow of liquid nitrogen inside UHV AFM cryostat. This rapid cooling process freezes the domains with different work function terminating at grain boundaries or layer edges (Fig. 8), which is a state seemingly identical to the one after regular annealing process. Rapid quenching traps the system in a metastable state from which the sample has a difficult time to escape. We have observed the same area of the sample for extensive time, and the spatial distribution of the work function did not change for a time period of at least 7 days. Only after thermally assisting the equilibration process by heating to temperatures above 120 °C, the work function relaxed to its original equilibrium value as in the previous cases. This is possibly an indication that the disordered structure that is in a metastable state far from the equilibrium needs prolonged exposure to elevated temperatures, which would facilitate defect diffusion and agglomeration as a precursor to the transition to a homogeneous ground state.

## Conclusions

Using combination of scanning tunneling microscopy and spectroscopy, Kelvin probe force microscopy and scanning transmission electron microscopy we have investigated the nature of defects in monolayer and few-layer films of MoS_2_ grown by CVD. We identify the structural and electronic signature of equilibrium state defects. We find that the work function and local density of states values are strongly affected by nearby presence of grain boundaries and edges. Relatively low annealing temperatures create metastable defect distribution that slowly relaxes to equilibrium state. We observed presence of metastable domains with different work function values caused by mobile defects induced by sample heating. STEM images and general n-type characteristics of formed domains, their dynamics and time scales of the equilibration process indicate a sulfur vacancy diffusion process with activation energies of roughly 0.6 eV. As the reported effects are observed under conditions typical for sample preparation and processing, our work underscores the importance of defect control and passivation when fabricating high-performance dichalcogenide-based devices.

## Methods

### CVD Growth of MoS_2_ and sample preparation

MoS_2_ films were grown using ambient pressure chemical vapor deposition technique using ultra-high purity N_2_ (250 sccm) as the carrier gas. HOPG substrates were cleaved with scotch tape just prior to loading in the furnace. Substrates were suspended facedown above 15 mg of MoO _3_ (≥99.5% Sigma Aldrich) in a crucible placed downstream from a different crucible containing 80 mg of Sulfur (≥99.5% Sigma Aldrich). Each crucible was placed in a different heating zone in a 1” furnace. Temperatures in these two zones were individually controlled using two adjacent tube furnaces. The furnace containing the MoO _3_ and HOPG was degassed at 150 °C for 90 minutes then ramped to 700 °C at rate of 15 °C/min. Once this furnace reached 320 °C the furnace containing the Sulfur crucible was ramped to 120 °C at approximately 3 °C/min. Both furnaces were allowed to sit at their maximum temperatures for 30 minutes at which point the MoO_3_ furnace was ramped down at 8 °C/min. Once this furnace reached 580 °C both furnaces were rapidly cooled to room temperature.

All annealing of MoS_2_ on HOPG was done *in-situ* in the microscope UHV chamber by e-beam heating of the Cu sample holder. Reported temperatures were measured by thermocouple located at the sample.

### Scanning Tunneling Microscopy and Spectroscopy

Scanning tunneling microscopy and spectroscopy measurements were carried out using a Unisoku STM with a PtIr tip in an ultra-high vacuum (!10^−11^ Torr) at room temperature. Prior to measurements all samples were degassed at approximately 300 °C and 10^−10^ Torr for at least two and a half hours and either allowed to cool naturally or quenched rapidly to room temperature before being moved to the scanner without breaking the vacuum. The STM images were recorded in constant current mode with a tunneling current of between 30–150 pA. For the $\frac{dI}{dV}$ spectra a minimum of 50 I–V curves were acquired per point, averaged and the numerical derivative was taken to obtain the $\frac{dI}{dV}$ conductance spectra.

### Kelvin Probe Force Microscopy

Kelvin Probe Force Microscopy measurements were performed in UHV conditions (p = 10^−10^ Torr) at room temperature in an RHK UHV-300 microscope with Nanonis BP4.5 controller. We employed single-pass FM-KPFM technique in non-contact mode with Pt/Ir coated tips (Pointprobe-Plus PPP-NCHPt-10; f_0_ = 360 kHz; k = 50 N/m) at constant oscillation amplitude, Δf set-point of −5 Hz and bias modulation amplitude of 810 mV at 238 Hz.

### TEM Sample Preparation

MoS_2_ films were grown on sapphire substrates and transferred to Quantifoil TEM grids using a dry transfer method^50^. First 9 g of polystyrene with a molecular weight of 280,000 g/mol was dissolved in 100 mL of toluene. This polymer mixture was then spin coated on top of the films for 60 s at 3500 rpm. The samples were subsequently heated on a hot plate at 100 °C for 15 minutes. The film/polystyrene structure was then lifted from the substrate using a droplet of water to penetrate between the film and the substrate. This was accomplished by pushing the water droplet under the film with sharp tweezers until the film/polystyrene structure separated from the substrate. The water was then removed and the structure was placed on Quantifoil TEM grids and baked on a hotplate first at 80 °C for one hour followed by 150 °C for 30 minutes. The polystyrene was removed by washing in toluene, acetone and IPA several times each. The MoS_2_ on the grids were then baked to remove residual solvents at 90 °C for 15 minutes before being measured with TEM.

### Scanning Transmission Electron Microscopy

Annular dark field scanning transmission electron microscopy images were taken using the aberration corrected JEOL JEM-ARM200CF scanning transmission electron microscope. The microscope was operated at 80 keV accelerating voltage to minimize beam induced knock-on damage. To improve signal-to-noise ratio, medium-angle annular dark field detector was used to acquire STEM images. The ADF images were deconvoluted to eliminate the effects of the probe function and enhance the contrast.
